# Diagnostic Yield of Endoscopic Ultrasound-Guided Liver Biopsy in Comparison to Percutaneous Liver Biopsy: A Two-Center Experience

**DOI:** 10.3390/cancers13123062

**Published:** 2021-06-19

**Authors:** Antonio Facciorusso, Daryl Ramai, Maria Cristina Conti Bellocchi, Laura Bernardoni, Erminia Manfrin, Nicola Muscatiello, Stefano Francesco Crinò

**Affiliations:** 1 Gastroenterology Unit, Department of Medical and Surgical Sciences, University of Foggia, 71122 Foggia, Italy; antonio.facciorusso@virgilio.it (A.F.); nicomuscatiello@gmail.com (N.M.); 2 Department of Internal Medicine, The Brooklyn Hospital Center, Brooklyn, New York, NY 11201, USA; dramai@tbh.org; 3 Gastroenterology and Digestive Endoscopy Unit, The Pancreas Institute, G.B. Rossi University Hospital, 37134 Verona, Italy; mariacristina.contibellocchi@aovr.veneto.it (M.C.C.B.); laura.bernardoni@aovr.veneto.it (L.B.); 4 Department of Diagnostics and Public Health, G.B. Rossi University Hospital, 37134 Verona, Italy; erminia.manfrin@univr.it

**Keywords:** endoscopic ultrasound, liver biopsy, percutaneous liver biopsy

## Abstract

**Simple Summary:**

Traditionally, liver biopsy has been performed by percutaneous radiology-guided methods. Advances in endoscopic ultrasound have demonstrated the efficacy of endoscopic based techniques for liver biopsy. Studies comparing both methods are scarce and have conflicting results. Our study compares percutaneous and endoscopic ultrasound methods for liver biopsy. Our analysis shows no evidence to support the wide use of endoscopic ultrasound. Percutaneous liver biopsy remains the sampling method of choice in this field.

**Abstract:**

There is scarce and conflicting evidence on the comparison between endoscopic ultrasound (EUS) and percutaneous (PC)-guided liver biopsy (LB). The aim of this study was to compare the two approaches in a series of patients with parenchymal and focal liver lesions. Fifty-four patients undergoing EUS-LB in two high-volume centers between 2017 and 2021 were compared to 62 patients who underwent PC-LB. The primary outcome was diagnostic adequacy rate. The secondary outcomes were diagnostic accuracy, total sample length (TSL), number of complete portal tracts (CPTs), procedural duration, and adverse events. Variables were compared using the Chi-square and Mann–Whitney test. Median age was 56 years (interquartile range 48–69) in the EUS-LB group and 54 years (45–67) in the PC-LB group with most patients being male. Indication for LB was due to parenchymal disease in 50% of patients, whereas the other patients underwent LB due to focal liver lesions. Diagnostic adequacy was 100% in PC-LB and 94.4% in the EUS-LB group (*p* = 0.74), whereas diagnostic accuracy was 88.8% in the EUS-LB group and 100% in the PC-LB group (*p* = 0.82). Median TSL was significantly greater in the PC-LB group (27.4 mm, IQR 21–29) when compared to the EUS-LB group (18.5 mm, 10.1–22.4; *p* = 0.02). The number of complete portal tracts was 21 (11–24) in the PC-LB group and 18.5 (10–23.2) in EUS-LB group (*p* = 0.09). EUS-LB was a significantly longer procedure (7 min, 5–11 versus 1 min, 1–3 of PC-LB; *p* < 0.001) and no evidence of adverse events was observed in any of the study groups. These results were confirmed in the subgroup analysis performed according to an indication for LB (parenchymal disease versus focal lesion). Although PC-LB yielded specimens with greater TSL, diagnostic adequacy and accuracy were similar between the two procedures.

## 1. Introduction

Liver biopsy (LB) represents the gold standard in the diagnostic algorithm of several hepatic disorders and focal liver lesions. In fact, although non-invasive testing such as liver stiffness measurement through transient elastography and the improvement in diagnostic tools for focal liver lesions decreased the need for LB [1,2], histologic analysis remains necessary in the case of uncertain diagnoses or when immunohistochemistry is needed. 

LB has been commonly performed for years using percutaneous (PC-LB), under CT-scan or ultrasonographic (US) guidance, and transjugular (TJ-LB) approaches. However, even in high-volume centers, PC-LB might determine a non-negligible sampling error rate, thus decreasing diagnostic sensitivity of the procedure; furthermore, as usually only the right lobe is accessible for biopsy, focal lesions located in the left lobe could be less easily sampled. Conversely, TJ-LB represents a more complex procedure and potential complications are neck hematoma, vascular injury, arterio-venous fistula, and intra-abdominal hemorrhage [3]. 

EUS fine-needle aspiration (FNA) and fine-needle biopsy (FNB) are a well-established diagnostic approach and are being employed for years for targeting both focal lesions and parenchymal liver disease [4,5]. To this end, many studies evaluating different needles on random liver biopsies have found superior results with FNB compared to FNA [6].

A recent pooled analysis by Mohan et al. reported a histologic diagnostic rate of 93.9% and adverse event rate of 2.3% with EUS-guided LB, with better outcomes observed with 19G FNA needles [7]. However, the promising results observed with newer FNB needles, such as the Franseen needle (Acquire^®^ [Boston Scientific, Marlborough, MA, USA]) and the Fork-tip needle (SharkCore^®^ [Medtronic, Dublin, Ireland]), in tissue acquisition of pancreatic masses [8,9,10,11,12,13] and of subepithelial lesions [14] also require confirmation in the setting of liver biopsy. 

Recent studies provided discording evidence on the comparative efficacy of EUS-LB as compared to PC-LB to diagnose parenchymal liver disease. In fact, while previous retrospective studies showed similar results between these two approaches [15,16,17], a recent small randomized-controlled trial (RCT) suggested better diagnostic performance with PC-LB [18].

Furthermore, no evidence on the comparison between the two procedures for tissue sampling of focal liver lesions is available in the literature. 

The aim of our study was to compare EUS-LB and PC-LB in terms of diagnostic outcomes including accuracy and safety in a series of patients with both parenchymal and focal liver disease from two high-volume centers in Italy.

## 2. Materials and Methods

### 2.1. Patients

From a prospectively collected database of 65 patients already undergoing endoscopic ultrasound for different primary EUS indications who would also benefit from a liver biopsy as determined by the referring hepatologist (i.e., abnormal liver function test results, fatty liver disease or suspected focal liver lesions on imaging), data from 54 patients undergoing EUS-guided LB were retrospectively reviewed. Included patients were enrolled in two high-volume Italian centers between 2017 and 2021. Institutional Review Board (IRB) approbation for this retrospective report was obtained. 

The following exclusion criteria were used: (1) reluctance to receive EUS-LB or inability to sign informed consent; (2) clear indication for surgical treatment; and (3) coagulopathy (international normalized ratio > 1.5, platelets < 50,000); concurrent antiaggregant or anticoagulant therapy. 

All procedures were performed by two board-certified 10 years-experienced gastroenterologists (N.M. and S.F.C). 

To compare these EUS-LB cases with PC-LB, 62 patients with suspected liver parenchymal disease or focal liver lesions sampled through percutaneous approach at our Institutions between 2015 and 2020 were retrospectively identified. The aforementioned exclusion criteria were applied also to PC-LB patients. 

### 2.2. EUS-LB

EUS-LB was performed under general sedation with propofol (Diprivan^®^, AstraZeneca, London, UK) and EUS was conducted with a Pentax EG3870-UTK ultrasound endoscope (Pentax Europe, Ltd., Hamburg, Germany) using a curved-array transducer. No pre-procedure antibiotics were administered.

Once in the stomach, the liver was carefully explored and before puncture of the desired lobe, color Doppler imaging was used to ensure the lack of vascular structures or bile ducts in the expected trajectory of the needle. The right and left lobe of the liver were accessed by the transduodenal and transgastric approach, respectively.

Several FNB needles (22G ProCore^®^ [Cook Medical, Bloomington, IN, US], 22G SharkCore^®^, or 22G Acquire^®^) and 19G FNA (EchoTip Ultra^®^, Cook Medical LLC, Bloomington, IN, USA) were used; the choice of the needle was left to the individual operator, and in the case of focal liver lesions, the size of the needle was chosen according to the size and the depth of the lesion itself.

**Figure 1:**
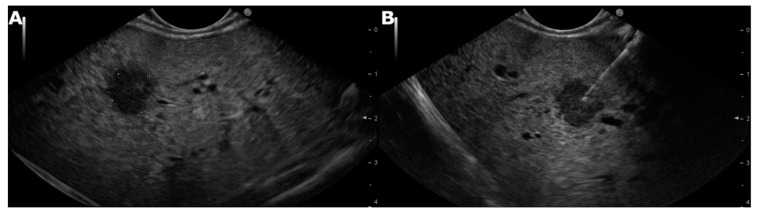
(**A**) Linear echoendoscope showing a focal lesion in the right lobe of the liver. (**B**) Linear echoendoscope showing fine-needle biopsy of the focal lesion in the right lobe of the liver.

**Figure 2:**
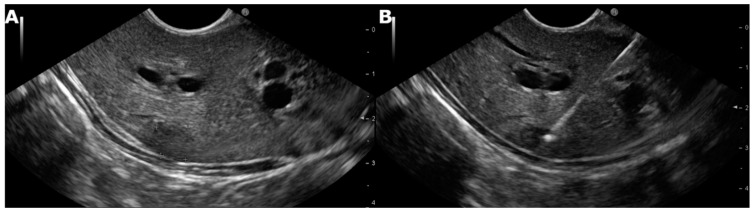
(**A**) Linear echoendoscope showing a focal lesion in the left lobe of the liver in segment II. (**B**) Linear echoendoscope showing fine-needle biopsy of the focal lesion in the left lobe of the liver in segment II.

Two passes were performed from either lobe of the liver in the case of parenchymal disease, whereas in patients with focal lesions, the need for additional passes was decided according to the macroscopic appearance of the collected material (Figure 1 and Figure 2).

Under EUS-guidance, the needle was advanced into the nodule/liver parenchyma and 10–20 to and from movements were performed while the stylet was slowly retrieved (slow-pull technique). 

Patients were continuously monitored during the procedure by a board-certified anesthesiologist with an automated non-invasive blood pressure device, electrocardiogram tracing and pulse oximetry to evaluate vital signs. Depending on complexity of the procedure and comorbidity, patients were either hospitalized for observation for 24 h or had the procedure in the day hospital. In both cases, the monitoring protocol was the same regardless of setting.

### 2.3. PC-LB

PC-LBs were performed with patients in supine position under local anesthesia. All percutaneous biopsies were performed after an injection of 2% lidocaine solution using a 25-gauge needle into the subcutaneous tissue and up to the liver capsule. No pre-procedure antibiotics were administered. A 16-gauge biopsy needle (Biopince^®^, Argon Medical Devices, Frisco, TX, USA) was inserted into the liver parenchyma under ultrasound-guidance. One core biopsy specimen was obtained, with a second pass performed only if the first pass yielded no specimen.

### 2.4. Outcomes

**Figure 3:**
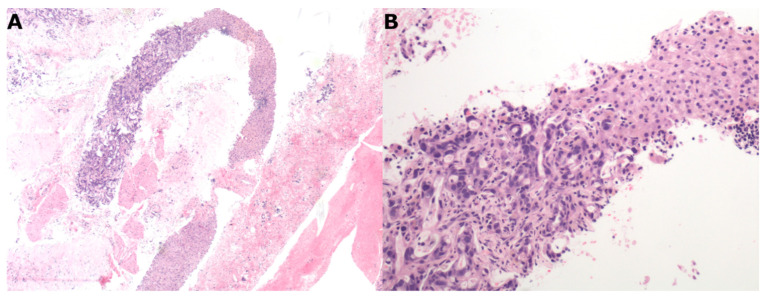
Liver metastasis from ductal adenocarcinoma of the pancreas. (**A**) 4 mm needle biopsy of a liver focal lesion with a representative area of adenocarcinoma occupying about 40% of the biopsy (upper left side) (4×, EE). (**B**) At higher magnification, adenocarcinoma cell aggregates (left side) replacing normal hepatocytes (right side) (20×, EE).

All specimens were collected in 10% formalin and sent to the laboratory for processing. The pathologist evaluating the LB specimen was blinded to the method adopted for performing LB (Figure 3). 

Primary outcome was diagnostic adequacy rate, defined as the proportion of patients deemed to have adequate samples for histological diagnosis. Secondary outcome was diagnostic accuracy, defined as true positive + true negative divided by total number of patients. The gold standard for diagnosis was considered surgery or clinical follow-up for at least 6 months. Given the absence of a reliable gold standard for diagnosis, patients undergoing LB due to parenchymal disease were excluded from the accuracy analysis. 

We also measured the total sample length (TSL) and the number of complete portal tracts (CPTs), where a CPT was defined as the presence of all 3 portal structures (portal vein, hepatic artery, and bile duct) in the sample. Procedural duration and procedure-related severe adverse events were also compared. For EUS-guided biopsy, procedural duration was defined as the time taken to procure the sample after identifying the lesion in the case of focal lesions, or as the time taken to procure two samples after identifying the left or right lobe of liver in the case of parenchymal disease. For the percutaneous biopsy method, this outcome was defined as the time from injection of lidocaine to successful procurement of the liver biopsy specimen.

### 2.5. Statistical Analysis

Categorical variables were reported as the number of cases and percentage, while differences between groups were compared using the Chi-square test. Continuous variables were expressed as median and interquartile (1st–3rd) range (IQR) and differences between groups were explored by the Mann–Whitney test. All analyses were 2-tailed, and the threshold of significance was assessed at <0.05. Subgroup analysis according to indication to LB (whether focal lesion or parenchymal disease) was performed. The statistical analysis was run using the MatchIt package in R Statistical Software 3.0.2 (Foundation for Statistical Computing, Vienna, Austria).

## 3. Results

### 3.1. Patients

During the study period, baseline characteristics of the whole study population of 116 patients who underwent liver biopsy are reported in Table 1. Fifty-four patients underwent EUS-guided LB (Group 1) and 62 underwent PC-LB (Group 2).

Indication to EUS in group 1 was diagnostic work-up of focal pancreatic masses in 28 patients (51.9%), characterization of pancreatic cystic lesions in 18 patients (33.3%), suspected autoimmune pancreatitis in 4 patients (7.4%), and sampling of upper GI subepithelial lesions in 4 patients (7.4%). 

As reported in Table 1, no difference was observed in the comparison between the two study groups. Median age was 56 years (IQR 48–69) in group 1 and 54 years (45–67) in group 2 (*p* = 0.24). Thirty-two (59.2%) patients in the EUS-LB group and 38 patients (61.2%) in the PC-LB group were male (*p* = 0.82), and indication to LB was due to parenchymal disease in 50% of patients whereas the other half of the sample size underwent LB due to focal liver lesions (*p* = 1.0). Median size of sampled focal lesions was 17 mm (11–22) and 18 mm (13–23) in the two groups, respectively (*p* = 0.83). 

Participants with liver cirrhosis was present in 27 patients in group 1 (50%) and in 38 patients (61.2%) in group 2 (*p* = 0.82). No difference was registered in terms of liver function tests (total bilirubin *p*= 0.45, aspartate aminotransferase *p* = 0.58, alanine aminotransferase *p* = 0.14, platelet count *p* = 0.33, international normalized ratio *p* = 0.73). 

Biopsy was performed in the right lobe of the liver in 17 patients with focal lesions (62.9%) in the EUS-LB group and in 19 patients (61.2%) in the PC-LB group (*p* = 1.0). Moreover, no difference in terms of liver segments sampled was observed (*p* = 0.89). 

In the EUS-LB group, 19G FNA was used in 29 patients, 25G ProCore^®^ in 1 patient, 22G SharkCore^®^ in 8 patients, 25G SharkCore^®^ in 11 patients, and 25G Acquire^®^ in 5 subjects. Parenchymal biopsies were performed using a 19G needle in 25 (92.6%) cases and a 22G needle in 2 (7.4%) cases. Focal lesions were sampled using a 19G, a 22G and a 25G needle in 4 (14.8%), 6 (22.2%), and 17 (63%) cases, respectively. A 25G needle was usually used to sample small focal lesions. The median number of passes was 2 (1–2) in EUS-LB group and 1 (1–1) in the PC-LB group. 

### 3.2. Outcomes

A detailed list of study outcomes is reported in Table 2. Diagnostic adequacy rate was slightly superior in the PC-LB group (100% vs. 94.4%) without reaching the significance threshold (*p* = 0.74). 

Median TSL was significantly greater in the PC-LB group (27.4 mm, IQR 21–29) compared to the EUS-LB group (18.5, 10.1–22.4; *p* = 0.02). The number of complete portal tracts was superior in the PC-LB group, although the difference only approached the significance threshold (21, 11–24 in the PC-LB group versus 18.5, 10–23.2 in the EUS-LB group; *p* = 0.09). 

Diagnostic accuracy rate in patients with focal liver lesions was 88.8% in the EUS-LB group and 100% in the PC-LB arm (*p* = 0.82). Final diagnosis was hepatocellular carcinoma in 28 patients (12 in group 1 and 16 in group 2), liver metastases in 24 patients (14 in the EUS-LB and 10 in the PC-LB group) and 41 patients were diagnosed with chronic hepatitis (17 in the EUS-LB and 24 in the PC-LB group). Non-alcoholic steatohepatitis was detected in 8 patients (4 in each group) whereas an absence of hepatic disease was registered in 15 subjects (7 in the EUS-LB and 8 in the PC-LB group). Overall, no difference in terms of pathologic conditions diagnosed was observed between the two groups (*p* = 0.74).

EUS-LB was a significantly longer procedure (7 min, 5–11 versus 1 min, 1–3 of PC-LB; *p* < 0.001) and no evidence of severe adverse events was observed in any of the study groups. 

### 3.3. Subgroup Analysis

As described in Table 3, results of the main analysis were confirmed in the subgroup analysis performed according to the indication to LB (parenchymal disease versus focal lesion). 

Median TSL was 10 mm (8.9–21.3) and 15 mm (11.3–25.8) in the EUS-LB and PC-LB group, respectively (*p* = 0.03), in patients with focal lesions whereas total sample length was 21.5 mm (14.1–25.4) and 30.2 mm (23–34) in the two groups, respectively (*p* = 0.008), in patients with parenchymal disease. 

Diagnostic adequacy was 96.2% in the EUS-LB group and 100% in the PC-LB group in the case of parenchymal disease (*p* = 0.88); likewise, adequacy rate was 92.2% in the EUS-LB and 100% in the PC-LB group in patients with focal lesions (*p* = 0.79). Moreover, EUS-LB resulted significantly longer than PC-LB in both subgroups (*p* < 0.001). 

## 4. Discussion

Liver biopsy represents the gold standard method for assessment of fibrosis severity in chronic liver disease [19]. Tissue sampling is used to determine the degree or stage of fibrosis. However, traditional percutaneous liver biopsy techniques are limited by the risk of potentially serious complications, inter-observer variations, and sampling errors, leading to false negative diagnosis [20,21]. Furthermore, percutaneous liver biopsy is associated with post-procedural pain. 

EUS-guided LB is well known to achieve optimal core histology samples. Furthermore, EUS-LB has the advantage of being able to obtain multiple liver passes quickly and safely from both lobes of the liver. 

This decreases the histologic variability through sampling different areas of the liver in addition to providing imaging assessment of other intra-abdominal organs.

However, there is still no consensus on what an ‘adequate’ liver biopsy actually represents. AASLD guidelines suggest that adequate liver biopsy specimens be at least 1.5 cm in length and contain more than 11 portal tracts. The Royal College of Pathologists defines adequacy as being greater than 1 cm in length and containing at least 6 portal tracts [22,23]. 

Although preliminary studies have not shown any significant difference between EUS-LB and PC-LB, a recent RCT found a clear superiority of PC-LB over EUS-LB in terms of optimal core procurement based on the aforementioned criteria [18]. This randomized clinical trial demonstrated that the PC-LB method yielded significantly more optimal specimens, defined as specimens with a length of 25 mm or greater and the presence of at least 11 complete portal tracts (CPT), compared with the EUS-guided method (57.9% vs. 23.8%, *p* = 0.028). While the PC-LB was associated with greater post-procedural pain, it was less costly (US$1824 vs. US$3240, *p* < 0.001) [18].

Currently, the 16G Biopince^®^ needle is the standard-of-care device for performing PC biopsies. It integrates a triaxial core, cut and capture system with an automated firing sequence. This enables the operator to procure of a full core of tissue alongside the whole diameter and length of the needle (full core biopsy needles). On the contrary, large calibre EUS–FNB needles are not larger than 19 G in diameter. These needles’ geometries are Franseen, Fork-tip, Menghini or Reverse bevel in design, and have two to three cutting edges or side holes at the tip to facilitate core tissue procurement [24,25]. Therefore, the EUS–FNB devices are not full core biopsy needles. 

Additionally, compared with transgastric or transduodenal biopsies where the FNB needle tip is partially flexed as it is moves back and forth in different trajectories within a target organ, the PC method uses a single cut motion in a straight plane. These differences were likely responsible for the more favorable diagnostic outcomes of PC-LB, compared to the EUS-LB in the aforementioned trial [18].

Therefore, given the scarce and conflicting evidence on this topic, we decided to perform a retrospective analysis of our series of patients who underwent to EUS-LB and PC-LB in two high-volume Italian centers. 

Diagnostic adequacy was slightly superior in PC-LB group (100% vs. 94.4%), although this difference was not statistically significant (*p* = 0.74), in accordance with previously published studies [18,26]. Our results confirmed the findings of the aforementioned trial with a greater median TSL in the PC-LB group (27.4 mm as compared to 18.5 mm in the EUS-LB group; *p* = 0.02). 

Similarly, the number of complete portal tracts was superior in the PC-LB, although the difference only approached the significance threshold (*p* = 0.09); the limited sample size in our study may have contributed to a lack of statistical significance and the trend in favor of PC-LB should be confirmed in larger clinical series. 

It is worth noting that the current manuscript represents the first comparative series recruiting patients with focal liver lesions, who represented half of the sample size. Subgroup analysis confirmed the above reported results in both subsets of patients; therefore, we can conclude that PC-LB provides higher quality samples even with comparable accuracy and adequacy rates both in patients with parenchymal disease and in the case of focal liver lesions. 

Accuracy rates were favorable with both techniques regardless of the final diagnosis, thus pointing out the striking performances of both PC-LB and EUS-LB in several hepatic pathological conditions. As expected, EUS-LB was a significantly longer procedure (7 min versus 1 min; *p* < 0.001), a further drawback of this technique compared to PC-LB. However, EUS-LB should not compete with PC-LB but play a complementary role and should be considered when EUS is already indicated for different reasons. Thus, patients could benefit from two high-quality evaluations during a single procedure. Moreover, in cases of small liver focal lesions located in positions which are difficult to reach percutaneously, EUS-LB could be considered to be a first line procedure.

Finally, none of the recruited patients experienced serious adverse events with EUS-LB or with the percutaneous approach. 

This study has several strengths: first, it is the first series directly comparing EUS-LB and PC-LB in patients with focal liver lesions, unlike previous studies that enrolled only patients with parenchymal liver diseases. Second, all the main diagnostic outcomes were collected with specific pathological parameters such as TSL and the number of CPTs. Third, the multicentricity of the current study allows a reliable reproducibility of our findings.

Nevertheless, our study has some weaknesses. Its main limitation is the retrospective nature of the study which could have led to selection biases. However, the two groups were comparable in terms of all the main baseline parameters; thus, the study groups were perfectly balanced without statistically different clinical and demographic features. In addition, cost considerations were beyond the scope of the present study and could not be addressed. However, it is hypothesized that the cost of EUS-LB may be substantially reduced when coupled with other endoscopic procedures (i.e., diagnostic or surveillance endoscopy).

Furthermore, it should be noted that the invasive and costly nature of LB along with the risk of adverse events have been responsible for a steady decline in the need for LB in patients with chronic liver disease and focal lesions. In fact, non-invasive measurements of liver stiffness are reliable and robust for assessing liver fibrosis/cirrhosis. These non-invasive methods allow for multiple and repeated assessments which can be safely performed in order to track the kinetics of liver stiffness after viral eradication in HCV patients [27]. 

Likewise, current guidelines restrict the need for LB in patients with focal lesions only in the case of inconclusive imaging findings [28,29]. Therefore, the role of LB remains uncertain in several settings; nevertheless, particularly in the case of focal liver lesions, the need for precise definition of specific biomarkers might reverse this trend and call for more LBs in the future [30,31,32]. 

## 5. Conclusions

EUS-LB is an alternative to percutaneous methods for liver biopsy. Our analysis provides robust evidence on the comparison between EUS-LB and PC-LB in patients with parenchymal liver disease and focal liver lesions. Therefore, based on our findings and the results of previous studies, there is no evidence supporting a wide use of EUS-LB. EUS-LB does not appear to be superior to PC-LB, and thus, PC-LB remains the sampling method of choice in this field. Broad prospective randomized trials with cost effective analyses are warranted in order to confirm the results of our analysis.

## Figures and Tables

**Table 1:** Baseline Patient Characteristics.

	GROUP 1 EUS-Guided Liver Biopsy (54 pts)	GROUP 2 Percutaneous Liver Biopsy (62 pts)	*p* Value
Age (years)			0.24
median (IQR)	56 (48, 69)	54 (45, 67)	
Gender			0.82
Male	32 (59.2%)	38 (61.2%)	
Female	22 (40.8%)	24 (38.8%)	
Biopsy			1.0
Focal lesion	27 (50%)	31 (50%)	
Parenchymal disease	27 (50%)	31 (50%)	
Lesion size (mm) *			0.83
Median (IQR)	17 (11–22)	18 (13–23)	
Liver cirrhosis			0.82
yes	27 (50%)	38 (61.2%)	
no	27 (50%)	24 (38.8%)	
Total bilirubin (mg/dL)			0.45
median (IQR)	0.7 (0.5–1.4)	1.1 (0.5–1.5)	
Aspartate aminotransferase (UI/dL)			0.58
median (min, max)	31.3 (15–260)	42.4 (25–302)	
Alanine aminotransferase (UI/dL)			0.14
Median (min, max)	44.3 (21–320)	52.7 (28.3–401)	
Platelet Count (×10^9^/L)			
Median (min, max)	210 (133–301)	298 (124–322)	0.33
International normalized ratio			0.73
Median (min, max)	0.9 (0.7–1.2)	1.2 (0.9–1.3)	
Site of liver biopsy *			1.0
Right lobe	17 (62.9%)	19 (61.2%)	
Left lobe	10 (37.1%)	12 (38.8%)	
Liver segments *			*p* value: 0.89
II	4	5	
III	6	7	
IV	5	5	
V	5	6	
VI	6	6	
VII	0	1	
VIII	1	1	
EUS Needle			--
19G FNA	29	--	
25G ProCore^®^	1		
22G SharkCore^®^	8		
25G SharkCore^®^	11		
25G Acquire^®^	5		

Values are expressed as number (percentage) or median (interquartile ranges) where specified; * Patients with focal lesions; Abbreviations: EUS, Endoscopic Ultrasound; IQR, Interquartile Range.

**Table 2:** Study outcomes.

	GROUP 1 EUS-Guided Liver Biopsy (54 pts)	GROUP 2 Percutaneous Liver Biopsy (62 pts)	*p* Value
Total length of specimen (mm)			**0.02**
median (IQR)	18.5 (10.1–22.4)	27.4 (21–29)	
No. of complete portal tracts			0.09
Median (IQR)	18.5 (10–23.2)	21 (11–24)	
Diagnostic adequacy			0.74
	51 (94.4%)	62 (100%)	
Diagnostic accuracy *			0.82
	24 (88.8%)	31 (100%)	
Final diagnosis			0.74
Hepatocellular carcinoma	12	16	
Liver metastasis	14	10	
Chronic hepatitis	17	24	
NASH	4	4	
Normal liver	7	8	
Procedural duration (mins)			**<0.001**
median (min, max)	7 (5–11)	1 (1–3)	
Procedure-related severe adverse events			1.0
	0 (0%)	0 (0%)	

Values are expressed as number (percentage) or median (interquartile ranges) where specified; * Patients with focal lesions; Abbreviations: EUS, Endoscopic Ultrasound; IQR, Interquartile Range. The bold indicates results with statistical significance.

**Table 3:** Subgroup analysis according to indication to biopsy (parenchymal disease versus focal lesion).

Variable	Subgroup	GROUP 1 EUS-Guided Liver Biopsy (54 pts)	GROUP 2 Percutaneous Liver Biopsy (62 pts)	*p* Value
Total length of specimen (mm)	Parenchymal disease	21.5 (14.1–25.4)	30.2 (23–34)	**0.008**
	Focal lesion	10 (8.9–21.3)	15 (11.3–25.8)	**0.03**
No. of complete portal tracts	Parenchymal disease	18.5 (10–23.2)	21 (11–24)	0.09
	Focal lesion	--	--	--
Diagnostic adequacy	Parenchymal disease	26 (96.2%)	31 (100%)	0.88
	Focal lesion	25 (92.2%)	31 (100%)	0.79
Diagnostic accuracy	Parenchymal disease	-	--	--
	Focal lesion	24 (88.8%)	31 (100%)	0.82
Procedural duration (mins)	Parenchymal disease	7 (5–10)	1 (1–3)	**<0.001**
	Focal lesion	7 (5–12)	1 (1–4)	**<0.001**

Values are expressed as number (percentage) in the case of categorical outcomes or median (interquartile ranges) in the case of continuous variables; Abbreviations: EUS, Endoscopic Ultrasound. The bold indicates results with statistical significance.

## Data Availability

The datasets used and/or analyzed during the current study are available from the corresponding author upon request.
